# LSTM-DGWO-Based Sentiment Analysis Framework for Analyzing Online Customer Reviews

**DOI:** 10.1155/2023/6348831

**Published:** 2023-02-11

**Authors:** Kousik Barik, Sanjay Misra, Ajoy Kumar Ray, Anthony Bokolo

**Affiliations:** ^1^JIS Institute of Advanced Studies & Research, JIS University, Kolkata, India; ^2^Department of Computer Science and Communication, Østfold University College, Halden, Norway; ^3^Department of Applied Data Sciences, Institute for Energy Technology, Halden 1777, Norway

## Abstract

Sentiment analysis furnishes consumer concerns regarding products, enabling product enhancement development. Existing sentiment analysis using machine learning techniques is computationally intensive and less reliable. Deep learning in sentiment analysis approaches such as long short term memory has adequately evolved, and the selection of optimal hyperparameters is a significant issue. This study combines the LSTM with differential grey wolf optimization (LSTM-DGWO) deep learning model. The app review dataset is processed using the bidirectional encoder representations from transformers (BERT) framework for efficient word embeddings. Then, review features are extracted by the genetic algorithm (GA), and the optimal review feature set is extracted using the firefly algorithm (FA). Finally, the LSTM-DGWO model categorizes app reviews, and the DGWO algorithm optimizes the hyperparameters of the LSTM model. The proposed model outperformed conventional methods with a greater accuracy of 98.89%. The findings demonstrate that sentiment analysis can be practically applied to understand the customer's perception of enhancing products from a business perspective.

## 1. Introduction

With the evolution of technology, business owners are releasing applications with different functionalities. Mobile apps are software-based applications installed on smartphones to offer a user-friendly experience. Different mobile applications, including entertainment, education, and business, have been released. Due to the proliferation of mobile applications, people are now shopping online through apps downloaded to their mobile devices instead of traditional web browsers. Approximately 66% of smartphone users utilize mobile apps [1]. Businesses make an effort to make their applications effective, convenient to use, and error free. They keep improving user experiences and application services by adding new features and functions [2]. App developers need a way to successfully compile user feature requests, feedback, and general thoughts to satisfy user needs [3]. Apps seek user ratings as an input to provide new services and enhance existing ones. Users can rate items using stars and provide text reviews for products. However, reviews include enormous amounts of unstructured information that cannot be manually evaluated.

**Figure 1:**
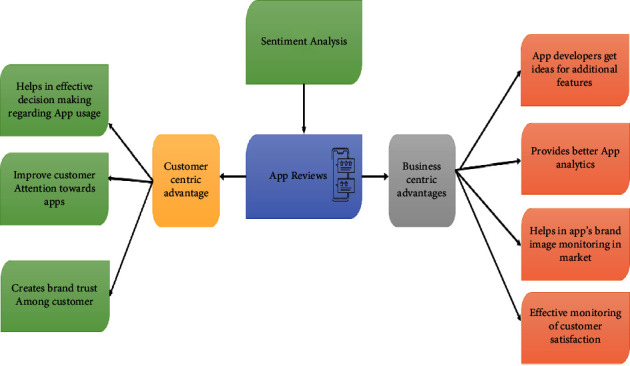
Advantages of sentiment analysis of app reviews.

Sentiment analysis (SA) is a field that analyses how customers react to products and services. Additionally, it measures how these sentiments are expressed in their attitudes and assessments. The connections between SA and product design still need to be explored. The SA's primary objective is to determine the polarity of the product on web commerce. As a result, it identifies the emotional state and uncovers the subjective data concealed in user experiences, and analyzing these sentiments in light of user feedback is crucial. Sentiment analysis and text mining need to be more consistent in driving suitable decisions, improving market competitiveness, and building customer trust [4]. The advantages of app review sentiment analysis are shown in Figure 1. Online reviews influenced 90% of consumer choices [5]. Most consumers choose the option with a high star rating because they believe it to be supported by favorable evaluations. However, the ratings provided by people on Internet platforms do not necessarily correspond to written reviews. The intelligent sentiment analysis (SA) system enables app developers to customize the products as per the requirements and interests of the customers.

The overall accuracy of existing research on sentiment analysis in polarity identification signs of progress can be improved by theoretical and technological difficulties [6]. The naive Bayes (NB), support vector machine (SVM), maximum entropy (MaxEnt), random forest (RF), and conditional random field frameworks are instances of conventional machine learning (ML) techniques that are often employed in sentiment analysis. Word2Vec, Glove, and FastText are some approaches that can automatically extract feature vectors from the text. However, the typical ML method still requires human communication to extricate the emotional aspects of the data from the input text [7]. Due to computational complexity and the selection of incomplete feature vectors in sentiment analysis, these ML techniques showed lower accuracy [8].

Sentiment analysis has made extensive use of deep learning (DL). Deep learning requires more processing power and storage than traditional ML algorithms since it uses more hidden layers, but accuracy has considerably improved. DL designs, including recurrent neural networks (RNNs), convolutional neural networks (CNNs), and gated recurrent unit (GRU) architectures, have been successfully used in text mining applications [9]. RNNs are helpful for many text-processing applications, but they encounter disappearing and expanding gradients when the input data contain long-term dependencies [10]. LSTM, however, improved sentiment analysis over RNN.

When a consumer accesses an online platform for purchase, they first review the feedback left by other customers. Based on that, the consumer makes a purchase decision. To improve the quality of customer service, many organizations are turning to the same problem-solving techniques [11]. However, the effectiveness of DL techniques for sentiment relies on how textual statements are characterized. The large dimensionality and sparsity of feature vectors resulting from traditional text representation techniques like bag-of-words and term frequency approaches reduce the accuracy of sentiment analysis. Therefore, a compelling feature extraction and selection approach is needed to raise the accuracy of sentiment analysis [12]. Deep learning optimization approaches have been used in a limited number of studies on user sentiment analysis for mobile apps. The significant contribution of the work is as follows:

1. The BERT model has been employed to obtain efficient word embeddings and automatic labels for online reviews in the preprocessing stage due to the quality of labeling being critical for efficient learning.
2. A genetic algorithm-based feature extraction method extracts suitable features from word embeddings, and the firefly algorithm-based feature selection method is used to obtain the optimum app review feature subset, which enhances the sentiment analysis process.
3. A deep neural network framework has been proposed to process online application reviews in a scalable way without affecting performance. An optimized deep learning technique, i.e., the LSTM-DGSO, is applied to classify the reviews into multiclasses like positive, negative, and neutral.
4. A comparative analysis has been presented and illustrates how the proposed model can be effectively used for sentiment analysis.

The remaining paper is formulated as follows: Section 2 discusses the related works. The proposed work, dataset, and techniques are illustrated in Section 3. The results and discussions are shown in Section 4. Finally, the paper is concluded in Section 5 with future work directions.

## 2. Literature Review

This section deals with recent studies conducted on online customer reviews using ML and DL approaches.

### 2.1. Machine Learning-Based Sentiment Analysis of Customer Reviews

Xia et al. [13] presented a conditional random field technique to extricate the emotional cues and SVM to identify the sentiment polarity of the reviews for classifying online reviews. The asymmetrical weighting of features used in this approach resulted in inconsistent accuracy. Tang et al. [14] presented the maximum entropy-based joint aspect-dependent sentiment topic approach (MaxEnt-JABST) to increase accuracy and performance in extracting aspects and opinions of online reviews. It has concerns with sentiment analysis across various domains to increase accuracy and performance in extracting aspects and opinions of online reviews. Shah et al. [15] proposed a novel strategy that included nine abstract-level dynamic analyses of user reviews using POS tagging and n-gram classification, followed by classification using NB and MaxEnt classifiers. However, the performance of sentiment analysis needs improvement for sentiment classification. Saad et al. [16] used multinomial logistic regression (MNB), support vector regression (SVR), decision trees (DTs), and RF algorithms. Jiang et al. [17] integrated SVM with IPSO (improved particle swarm optimization) to categorize attitudes.

### 2.2. Deep Learning-Based Sentiment Analysis of Customer Reviews

Chen et al. [18] proposed the deep belief network and sentiment analysis (DBNSA) to analyze user reviews and enhance user rating categorization. The categorization of user ratings by DBNSA involves complicated computing operations. A neural network model that includes the extraction of user behavior data from tweets was suggested by the authors of [19]. Asghar et al. [20] designed Senti-eSystem, a tool for assessing customer satisfaction using a hybrid fuzzy and deep neural network. Performance suffers due to the imbalance in the dataset gathered for this study. The multichannel convolution and bidirectional GRU multihead attention capsule (AT-MC-BiGRU-capsule) is a model for text sentiment analysis that replaces scalar neurons with vector neurons and employs capsules to define text emotions, as presented in [21]. The model lacks stability issues. Zulqarnain et al. [22] presented a two-state GRU (TS-GRU) depending on the feature attention process, focusing on word-feature capturing and sequential modeling to discover and classify the sentiment polarity. The TS-GRU approach could be more computationally challenging. Alam et al. [23] suggested a domain-specific distributed word representation with a dilated CNN for social media SA to create smart city apps but considered one domain. RNN was utilized in [24] to anticipate client opinions based on web reviews. Glove feature extraction produced unsatisfactory results when combined with the RNN algorithm. A hybrid deep CNN and LSTM models were suggested by the authors of [25] in the e-commerce industry. However, this strategy uses more computing resources. The best aspects from the online review were extracted by the authors of [26] using ML methods, and the selected features were subsequently put into the CNN for sentiment analysis. The suggested approach flexibility and computational efficiency have remained the same.

### 2.3. Sentiment Analysis of App Reviews

Using a latent Dirichlet allocation (LDA) model to find sentiments and a logistic regression model to determine the variables influencing E-rider satisfaction, Aman et al. [27] presented the app store comments from two prominent micromobility businesses. A biased allocation was produced by the LDA model that overemphasized topics relating to user experience and app performance. Rahman et al. [28] utilized ML classifiers, including K-nearest neighbor (KNN), RF, SVM, DT, and NB, together with NLP-based approaches like N-gram, bag-of-words, and TF-IDF. They discovered and built a well-fitted model to recognize user opinions on mobile applications. Aslam et al. [29] proposed the CNN-based DL methodology to categorize app reviews. However, this technique has yet to look at location-based and temporal traits. Jha et al. [30] suggested an improved dictionary-based multilabel classification method to categorize nonfunctional needs in user comments taken from samples of Android and iOS applications. However, the accuracy of this method could have been higher. Venkatakrishnan et al. [31] used an improved dataset to use NB, XGBoost, and multilayer perceptron (MLP) to examine the numerous app-centric variables and predict user ratings. The functionality of the model needs to be enhanced. Rustam et al. [32] used logistic regression, RF, and AdaBoost classifier approaches to categorize the reviews of Shopify applications. Due to traditional feature selection approaches, lower accuracy is obtained. RF, SVM, and NB were built for sentiment analysis of English textual comments obtained from three digital payment apps [33]. These techniques need to be more accurate in classifying emotions and could have been more cost effective. Tchakounte et al. [34] presented a model using NB to get information valuable for enhancing the security features of mobile applications. With differences in time, the findings could be more consistent.

Ireland et al. [35] employed logistics to demonstrate that sentiment classification of user-generated big data might be utilized to compare airline service quality to existing survey-based methods by analyzing real-time customer views. In particular, the article will look at how user-generated big data sentiment analysis might be utilized to study airline service quality. Oyebode et al. [36] presented a method to evaluate health record data using machine learning approaches. Lin et al. [37] offered a sentiment analysis model of app reviews using deep learning. Bose et al. [38] proposed a model using the NRC emotion lexicon and used six product reviews. They presented how sentiment analysis assists in determining the consumers' behaviors.

Zaki et al. [39] proposed a methodology for determining the significant labels denoting customer sentiments. The titles of the comments, which typically include the terms that most effectively characterize the customer experience, were combed through to locate relevant labels. The findings indicate that the labels developed from the titles are valid for analyzing the feelings expressed in the comments. Iqbal et al. [40] suggested that predicting attitudes demonstrates superior, or at the very least comparable, outcomes with much reduced computational complexity. The findings of this study highlight the critical significance of performing sentiment analysis on the content of consumer reviews and social media platforms to acquire valuable insights. Akram et al. [41] suggested a technique for short clustering text using a deep neural network. This approach learns clustering aims by transforming the high-dimensional feature space into a lower-dimensional feature space. Abbasi et al. [42] proposed a new method for authorship detection that combines ensemble learning, DistilBERT, and more traditional machine learning strategies. A count vectorizer and bigram term frequency-inverse document frequency (TF-IDF) are used in the suggested method to extract essential qualities. Witte et al. [43] offered an international survey for online consultations in mental health care using statistical analysis. Assaker et al. [44] proposed a model for the traveler using online travel reviews through an extended unified theory of acceptance and use of technology. This analysis improves the interpretation of the explicatory variables for online reviews. Assaker [45] presented the effects of trustworthiness and expertise on usage intention toward user-generated content and online reviews among female, male, younger, and older travelers.

Machine learning techniques like a binary support vector machine cannot be the most effective categorization when analyzing online customer reviews. Clustering, classification, regression, and rule extraction are some of the machine learning issues. Consequently, deep learning algorithms are used to analyze customer evaluation sentiments effectively, and the LSTM framework is effective for sentiment analysis. However, building an LSTM model with optimum hyperparameters is a complex problem. The application of the LSTM framework with optimum hyperparameters in sentiment analysis of online app reviews has yet to be explored. This motivates us to research the LSTM-DGSO methodology in sentiment analysis of customer reviews. The findings illustrate the detailed analysis performed, explore hidden factors of customer sentiment analysis, and build a model. A summary of current sentiment analysis models for online customer reviews is illustrated in Table 1.

## 3. Proposed Work

**Figure 2:**
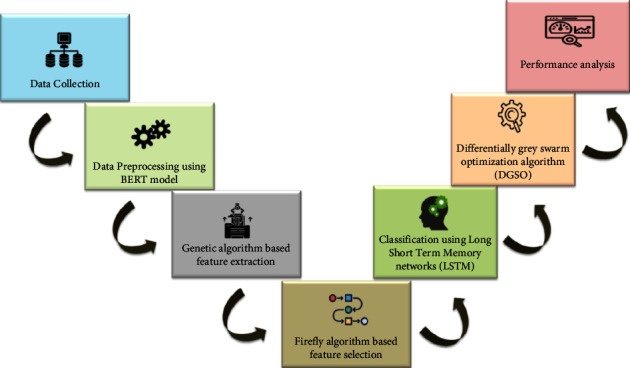
Proposed model for sentiment analysis of app reviews.

Discovering the sentiment class of reviews is a multiclass classification issue. Efficient and automatic classification of the reviews posted by app users into three classes, such as positive, negative, and neutral, is the main objective of this study.  Figure 2 depicts the overall framework for sentiment analysis of app reviews. Initially, we collected online reviews for Shopify apps from the Kaggle website. Then, the BERT model processed the reviews to obtain efficient word embeddings and extract labels for reviews whether they belong to positive, negative, or neutral classes. GA extracted more relevant app review features, and the optimum feature subset was obtained using FA. We employed the LSTM model to categorize the reviews. To improve the behavior of LSTM in sentiment analysis of reviews, hyperparameters of LSTM, like the learning rate and batch size, are optimized by the DGSO algorithm.  Figure 2 illustrates the proposed framework for sentiment analysis.

### 3.1. Data Collection

The Shopify app store dataset is utilized in this study and collected from Kaggle [46], and 50140 reviews are selected randomly. The dataset has eight fields; their description is presented in Table 2.

### 3.2. Data Preprocessing Using the BERT Model

**Figure 3:**
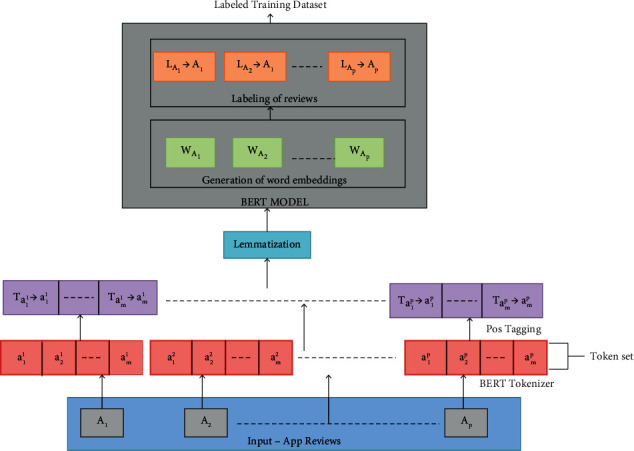
BERT model-based app review preprocessing.

In the preprocessing stage, the BERT model is used to represent app reviews efficiently and extract their labels. BERT is a compelling architecture that utilizes transformer-based topologies and is built on an encoder-decoder network [47], and the task-specific layers of the BERT model are crucial [48]. The steps involved in review preprocessing using the BERT model are demonstrated in Figure 3.

The document containing app reviews is provided as an input to the BERT model using the following equation:

(1) $$\begin{array}{c} \left[A\right]=\left\{A_{1},A_{2},\ldots .,A_{p}\right\}\text{,} \end{array}$$

where [*A*] denotes the app review set and *p* denotes the number of app reviews.

The preprocessing steps include removing the special characters and numerical data from app reviews and substituting uppercase characters with lowercase characters. The BERT model uses a WordPiece tokenizer to split the review sentences into a list of tokens or words. The token set for the app reviews obtained after tokenization is defined by equation (2). Then, stop words like prepositions, articles, and conjunctions are removed from the token set.

(2) $$\begin{array}{c} \left[A\right]=\left\{\left\{a_{1}^{1},a_{2}^{1},\ldots ,a_{m}^{1}\right\},\left\{a_{1}^{2},a_{2}^{2},\ldots ,a_{m}^{2}\right\},\ldots ..,\left\{a_{1}^{p}{,a}_{2}^{p},\ldots ,a_{m}^{p}\right\}\right\}\text{,} \end{array}$$

where *a*_*m*_^*p*^ denotes the mth token of the pth review and *m* denotes the number of tokens for each review.

The part of speech to which each token belongs is tagged to each token by POS (parts-of-speech) tagger. The POS-tagged app review vector is defined by the following equation:

(3) $$\begin{array}{c} \left[A\right]=\left\{\left\{{t_{a_{1}^{1}}⟶a}_{1}^{1},\ldots ,{t_{a_{m}^{1}}⟶a}_{m}^{1}\right\},\left\{{t_{a_{1}^{2}}⟶a}_{1}^{2},\ldots ,{t_{a_{m}^{2}}⟶a}_{m}^{2}\right\},\ldots ..,\left\{{t_{a_{1}^{p}}⟶a}_{1}^{p},\ldots ,{t_{a_{m}^{p}}⟶a}_{m}^{p}\right\}\right\}\text{,} \end{array}$$

where *t*_*a*_*m*_^*p*^_ denotes the POS tag assigned to the token and *a*_*m*_^*p*^ denotes the tag symbol.

Following POS tagging, the lemmatization process takes place to obtain token root words (lemma). Then, the BERT model creates word embeddings for app reviews. Tokens with higher semantic similarity are similarly represented in the word embedding. It contains feature words for each app review. The sentiment score for each app review is calculated using the following equation:

(4) $$\begin{array}{c} {Sentiment\_score}_{A_{p}}=\frac{\sum_{i=1}^{m}S_{a_{i}^{p}}}{m}\text{,} \end{array}$$

where sentiment_score_*A*_*p*__ denotes the sentiment score for the pth app review (*A*_p_), *S*_*a*_*i*_^*p*^_ denotes the sentiment value for token *a*_*i*_^*p*^, and *m* refers to the number of tokens in the pth app review.

The BERT model extracts the label for each app review depending on the sentiment_score. If the sentiment_score for the review (*A*_p_) is greater than 0, the review is labeled as positive. If the sentiment_score for the review (*A*_p_) is less than 0, the review is labeled as negative. If sentiment_score for the review (*A*_p_) equals 0, the review is labeled as neutral. As a result, the BERT model obtains word embeddings with respective labels for the app review dataset.

### 3.3. Feature Extraction Using the Genetic Algorithm (GA)

Dimensionality reduction is needed to ease the sentiment classification process. The dimensionality reduction problem can be formulated as an optimization issue. We employed the GA to reduce review feature dimensions. The advantage of using a genetic algorithm is that it can solve complex problems using traditional methods. The GA seeks a transformed review feature set in a y-dimensional space that satisfies the optimization criteria given a set of x-dimensional input app review data. The classification accuracy is utilized to assess modified patterns. Word embeddings of app reviews are provided as an input to the feature extraction step and defined as

(5) $$\begin{array}{c} \left[W_{p\times x}\right]=\left\{\left\{k_{1}^{1},k_{2}^{1},\ldots ,k_{x}^{1}\right\},\left\{k_{1}^{2},k_{2}^{2},\ldots ,k_{x}^{2}\right\},\ldots ,\left\{k_{1}^{p},k_{2}^{p},\ldots ,k_{x}^{p}\right\}\right\}\text{,} \end{array}$$

where [W] is the word embedding and *k*_*x*_^*p*^ denotes the *x*^th^ feature of the *p*^th^ review.

**Figure 4:**
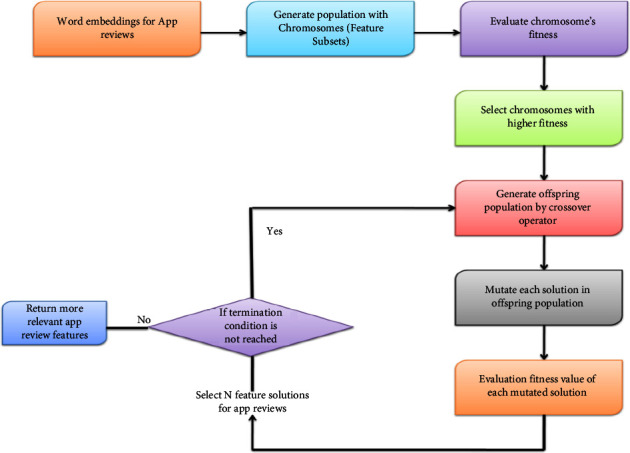
GA-based feature extraction from app review data.

Chromosomes make up the population in the GA. A solution vector is referred to as a chromosome or an individual in the GA. Genes are the separate building blocks that make up chromosomes. The procedure involved in GA-based feature extraction, as depicted in Figure 4 and Algorithm 1, is explained as follows. The population is randomly initialized with a set of chromosomes. Here, the GA randomly selects a set of features from the app review dataset and stores them in each chromosome for later usage. All features in chromosomes are encoded with real-number representations. If the x^th^ bit of the *i*^th^ vector equals 1, then the *k*_*x*_^*p*^*th* feature is permitted to take part in classification; if the bit is 0, then the corresponding feature does not take part in classification. Each resultant feature subset is rated based on categorization efficiency. The fitness value of each chromosome is evaluated by training the LSTM model over the feature subset and observing the classification accuracy. The fitness function for the GA is the number of properly identified app reviews given by the LSTM trained on the particular feature subset (chromosome). The fitness value of each chromosome in the population is determined by

(6) $$\begin{array}{c} {Fitness}_{i}=\frac{N_{correct}}{p}\text{,} \end{array}$$

where fitness denotes the fitness value of the *i*^th^ chromosome, *N*_correct_ denotes the number of correctly classified app reviews, and *p* denotes the number of app reviews to classify. Figure 4 shows GA-based feature extraction from review data.

The fitness value of each chromosome in the population is compared to the threshold fitness value. The chromosomes whose fitness value exceeds the threshold value are selected for the next generation or new solution generation. Crossover and mutations are the two operators that the GA uses to create new feature solutions from preexisting ones. In crossover, two chromosomes, referred to as parents, are typically combined to create new chromosomes, referred to as offspring. For the offspring to inherit excellent genes that make parents fitter, parents are chosen from the population's existing chromosomes with a preference for fitness. The offspring population containing new offspring is generated from chromosomes of the initial population, whose fitness value is greater than the threshold value. Then, each solution in the offspring population is mutated by a mutation operator with a specific mutation rate. The mutation operator modifies chromosomal properties at random. Usually, the mutation occurs at the gene (feature) level. Following mutation, the fitness value of each mutated offspring (modified feature solutions) in the offspring population is evaluated according to equation (6) using the LSTM model. Then, “*N*” mutated solutions that satisfy the condition that the fitness value of the solution must be greater than the threshold value are selected from the offspring population. Finally, the termination condition of the GA is checked. If the current iteration is less than the maximum iteration, the “*N*” mutated solutions obtained from the above step allow a new crossover and mutation operations. Up to the maximum repetition, the procedure above is repeated. If the current iteration is equal to the maximum iteration, the solutions containing more relevant features for sentiment analysis are obtained as a result of the GA. The *x*-dimensional input features of app reviews are transformed into a *y*-dimensional feature set.

### 3.4. Feature Selection Using the Firefly Algorithm

The optimum app review feature set that enhances the sentiment analysis performance must be selected from the *y*-dimensional app review feature set. The FA is utilized for choosing the optimum app review feature set in this paper. The pseudocode for FA-based feature selection is presented in Algorithm 2. A search space is initialized, having *y* dimensions corresponding to the features in the app review dataset. The search space is initialized with “*m*” number of fireflies. The position (*x*_*F*_*a*__) and intensity (*J*_*F*_*a*__) of each firefly in the search space are initialized. Each firefly at a specific position is represented as a binary vector with the “*y*” number of features for “*p*” app reviews and is denoted by

(7) $$\begin{array}{c} F_{a}=\left\{{\left\{k_{1}^{1},k_{2}^{1},\ldots ,k_{y}^{1}\right\}}_{F_{a}},{\left\{k_{1}^{2},k_{2}^{2},\ldots ,k_{y}^{2}\right\}}_{F_{a}},\ldots ..,{\left\{k_{1}^{p},k_{2}^{p},\ldots ,k_{y}^{p}\right\}}_{F_{a}}\right\}\text{,} \end{array}$$

where *a* = 1,…, *m*, *F*_*a*_ represents the *a*^th^ firefly representing the app review feature solution, *m* denotes the number of fireflies, and *k*_*y*_^*p*^ denotes the *y*^th^ feature of the *p*^th^ review.

Each element in *F*_*a*_ is limited to 0 or 1, indicating whether that app review feature is selected. If the *k*_*y*_^*p*^ feature is selected, then it is encoded as 1, and if it is not selected, it is encoded as 0. The change in brightness and attractiveness are two crucial aspects of the FA. Hence, the intensity of a firefly (*F*_*a*_) at a distance (*d*) from another firefly (*F*_*b*_) is defined by

(8) $$\begin{array}{c} J_{F_{a}}=J_{0}e^{-\delta \left(dd^{2}\left(F_{a}.F_{b}\right)\right)}\text{,} \end{array}$$

where *J*_*F*_*a*__ denotes the intensity of a firefly (*F*_*a*_) at a distance (*d*) from another firefly (*F*_*b*_), *J*_0_ denotes the initial brightness, *d*(*F*_*a*_.*F*_*b*_) refers to the distance between two fireflies, *F*_*a*_ and *F*_*b*_, and *δ* is the light absorption coefficient influencing intensity.

Depending on the intensity and distance, two fireflies, *F*_*a*_ and *F*_*b*_, are more or less attractive to one other. The attractiveness (*A*) of a firefly is determined by equation (9), proportional to the intensity noticed by another firefly.

(9) $$\begin{array}{c} A_{F_{a}}=A_{0}e^{-\delta {\left(d\right.}^{2}\left(F_{a}.F_{b}\right)}\text{,} \end{array}$$

where *A*_*F*_*a*__ denotes the attractiveness of a firefly (*F*_*a*_) at a distance (*d*) from another firefly (*F*_*b*_) and *A*_0_ denotes the attractiveness constant.

According to the classifier's efficiency using the chosen feature subset, each firefly travels in a specific direction in the search space to locate the ideal feature subset. Here, the LSTM model was used as an evaluating classifier. The correctness of the classifier using the chosen feature is regarded as the intensity of the firefly or objective function. The light intensity *J* of a firefly representing the app review feature subset is proportional to the fitness function according to the following equation:

(10) $$\begin{array}{c} J_{F_{a}}\alpha {Fitness}_{F_{a}}\text{.} \end{array}$$

The fitness function for the FA is defined by

(11) $$\begin{array}{c} {Fitness}_{F_{a}}=\frac{N_{correct}}{p}\text{.} \end{array}$$

Using equation (12), the firefly with a lower intensity (accuracy) will travel toward the firefly with a greater intensity (accuracy). When a firefly moves, its position and feature vector will change.

(12) $$\begin{array}{c} x_{F_{a}}^{t}=x_{F_{a}}^{t-1}+A_{F_{a}}e^{\delta {\left(d\right.}^{2}\left(F_{a}.F_{b}\right)}\left(x_{F_{b}}^{t-1}-x_{F_{a}}^{t-1}\right)+\gamma \left(r-\frac{1}{2}\right)\text{,} \end{array}$$

where *x*_*F*_*a*__^*t*^ and *x*_*F*_*a*__^*t*−1^ denote the position of the firefly *F*_*a*_ at time *t* and *t* − 1, respectively, *x*_*F*_*b*__^*t*−1^ denotes the position of the firefly *F*_*b*_ at time *t* − 1, *A*_*F*_*a*__ indicates the attractiveness of the firefly *F*_*a*_, *γ* denotes the randomization parameter, and *r* denotes a random number between 0 and 1. The distance between two fireflies, *F*_*a*_ and *F*_*b*_, is defined as

(13) $$\begin{array}{c} d\left(F_{a}.F_{b}\right)=\sqrt{{\left(x_{F_{a}}-x_{F_{b}}\right)}^{2}+{\left(y_{F_{a}}-y_{F_{b}}\right)}^{2}}\text{,} \end{array}$$

where (*x*_*F*_*a*__, *y*_*F*_*a*__) and (*x*_*F*_*b*__, *y*_*F*_*b*__) are the position vector of fireflies *F*_*a*_ and *F*_*b*,_ respectively, and *d*(*F*_*a*_.*F*_*b*_) denotes the distance between two fireflies, *F*_*a*_ and *F*_*b*_.

This process is repeated for other fireflies. A better feature solution at the end of the iteration will represent each firefly. This process is continued until the maximum iteration is reached. If the termination condition is achieved, all fireflies having the local best solutions are ranked depending on a fitness function. The highest intensity (accuracy) firefly is returned as the best global solution. As a result of the FA, the optimal app review feature set [*W*_optimal_] is obtained from the global best firefly.

### 3.5. Classification of App Reviews by the LSTM-DGSO Technique

The sentiment analysis includes classifying reviews into three classes: positive, negative, and neutral, using the LSTM-DGSO technique. In the LSTM-DGSO module, hyperparameters of the LSTM model, like the learning rate and batch size, are optimized by the DGSO technique. The optimized LSTM model is trained over the optimal app review feature subset. The procedure is shown in Algorithm 3. The first stage of the LSTM-DGSO module includes the optimization of hyperparameters of LSTM by DGSO. According to DGSO, grey swarm is generated with the “n” number of grey wolves in the search space. Grey swarm can be divided into four levels, namely, *α*, *β*, *γ*, and *δ*. The *α* grey agent is the head of the grey swarm, which controls the hunting, habitat, and moving behavior of grey swarm. The *β* grey agent is at the second level *γ*, and the grey agent obeys the commands *α* and *β* agents. The *δ* grey agent is the lowest agent in grey swarm. The number of maximum iterations is assumed to be *i*_max_. The hyperparameters of LSTM are initialized as R for the learning rate and B.S for the batch size. The position of each grey wolf represents the hyperparameter solution of LSTM. In each iteration, the grey wolf can search for prey in the search space. Here, the prey denotes the threshold app review classification accuracy. Based on the accuracy of the LSTM classifier with the given hyperparameters, each grey agent travels in a certain direction. An objective function is the classifier model's accuracy using the provided hyperparameters. In the search process, the position of each grey agent is changed continuously to achieve higher accuracy. The three wolves closest to the prey are automatically converted into the local best solutions of *α*, *β*, and *γ* grey agents. The position of each grey search agent according to three local best solutions at time *t* is updated using the following equation:

(14) $$\begin{array}{c} \overset{⟶}{V\left(t+1\right)}=\frac{\overset{⟶}{v_{1}}+\overset{⟶}{v_{2}}+\overset{⟶}{v_{3}}}{3}\text{,} \end{array}$$

where $\overset{⟶}{v_{1}}$, $\overset{⟶}{v_{2}}$, and $\overset{⟶}{v_{3}}$ are defined by the following equations:

(15) $$\begin{array}{c} \overset{⟶}{v_{1}}=\overset{⟶}{V_{\alpha }}-{\overset{⟶}{E}}_{1}.{\overset{⟶}{T}}_{\alpha }\text{,} \end{array}$$

(16) $$\begin{array}{c} \overset{⟶}{v_{2}}=\overset{⟶}{V_{\beta }}-{\overset{⟶}{E}}_{2}.{\overset{⟶}{T}}_{\beta }\text{,} \end{array}$$

(17) $$\begin{array}{c} \overset{⟶}{v_{3}}=\overset{⟶}{V_{\gamma }}-{\overset{⟶}{E}}_{1}.{\overset{⟶}{T}}_{\gamma }\text{,} \end{array}$$

where $\overset{⟶}{E}$ denotes the coefficient vector, defined by

(18) $$\begin{array}{c} \overset{⟶}{E}=2\overset{⟶}{b}.{\overset{⟶}{k}}_{1}-\overset{⟶}{b}\text{,} \end{array}$$

where $\overset{⟶}{b}$ is given by

(19) $$\begin{array}{c} \overset{⟶}{b}=2-t.\frac{2}{t_{\max}}\text{,} \end{array}$$

where *k*_1_ denotes the random vector and *t* and *t*_max_ denote the current and maximum iteration numbers, respectively.

${\overset{⟶}{T}}_{\alpha }$ , ${\overset{⟶}{T}}_{\beta },and{\overset{⟶}{T}}_{\delta }$ are determined by the following equations:

(20) $$\begin{array}{c} {\overset{⟶}{T}}_{\alpha }=\left|{\overset{⟶}{D}}_{1}.\overset{⟶}{V_{\alpha }}-\overset{⟶}{V}\right|\text{,} \end{array}$$

(21) $$\begin{array}{c} {\overset{⟶}{T}}_{\beta }=\left|{\overset{⟶}{D}}_{2}.\overset{⟶}{V_{\beta }}-\overset{⟶}{V}\right|\text{,} \end{array}$$

(22) $$\begin{array}{c} {\overset{⟶}{T}}_{\delta }=\left|{\overset{⟶}{D}}_{3}.\overset{⟶}{V_{\gamma }}-\overset{⟶}{V}\right|\text{,} \end{array}$$

where $\overset{⟶}{D}$ is the coefficient vector and is determined by

(23) $$\begin{array}{c} \overset{⟶}{D}=2{\overset{⟶}{k}}_{2}\text{.} \end{array}$$

**Figure 5:**
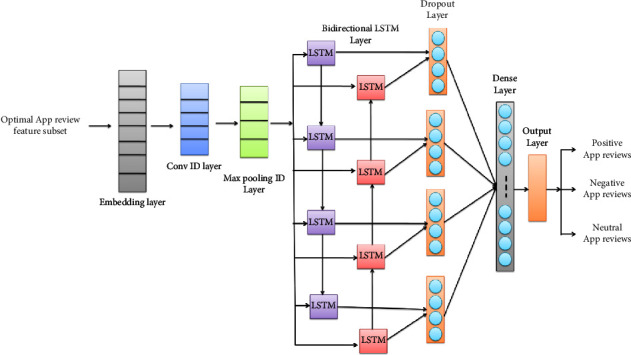
The proposed framework of the LSTM model.

Until the maximum loop is reached, the procedure above is repeated. The ideal hyperparameter solution for improving the sentiment analysis process by LSTM is derived via DGSO after the maximum number of iterations has been obtained. The optimal hyperparameters obtained from DGSO are set for the LSTM model. Our proposed LSTM model includes an embedding layer, a one-dimensional convolutional (Conv1D) layer, a one-dimensional max pooling (MaxPooling1D) layer, a bidirectional LSTM (Bi LSTM) layer, a dropout layer, and a dense layer. The framework of our proposed LSTM model is presented in Figure 5, and the architecture of the proposed LSTM model is shown in Table 3. The embedding layer efficiently represents the optimal app review feature set. The Conv1D layer generates a feature map for the selected app review features. There are 32 filters in the convolution layer with a kernel size of 3. The MaxPooling1D layer extracts the maximum information from the feature map. Then, the efficient feature map is sent as an input to the BiLSTM layer.

The feature sequences generated by the MaxPooling layer do not provide sequence information. With focus on sequential modeling, BiLSTM can further decode the feature sequences acquired by the previous layer to provide contextual information respective to app reviews. Both forward and backward LSTM units make up BiLSTM. Combining a forward hidden layer with a backward hidden layer allows BiLSTM to retrieve both the prior and subsequent contextual elements of app reviews. A group of memory blocks, often recurrently linked blocks, make up each LSTM layer. Each memory block contains memory cells and three gates: input, forget, and output. The process occurring in each LSTM unit is explained as follows. Figure 5 depicts the overall framework of the proposed LSTM model.

The feature map is added to BiLSTM neurons through activation function collaboration with the input gate. The forget gate's output has already been acquired at this gate. Equation (24) is used to calculate the output from the input gate.

(24) $$\begin{array}{c} x_{t}=\sigma \left(S_{wx}w_{t}+S_{rx}r_{t-1}+S_{dx}d_{t-1}+q_{x}\right)\text{,} \end{array}$$

where *x*_*t*_ denotes the output of the input gate at time *t*, *σ* is the logistic sigmoid function, *S*_wx_, *S*_rx_, and *S*_dx_ are the weight matrices for the input gate, *q*_*x*_ is the variable bias of the input gate, *W*_*t*_ is the information regarding app reviews at the time “*t*,” and *r*_*t* − 1_ and *d*_*t* − 1_ are the hidden and cell state at the time step t − 1, respectively.

The output of the preceding LSTM neuron can be used to modulate the forget gate of the present LSTM neuron. The output of the forget gate of the LSTM neuron is processed using the following equation:

(25) $$\begin{array}{c} g_{t}=\sigma \left(S_{wg}w_{t}+S_{rg}r_{t-1}+S_{dg}d_{t-1}+a_{g}\right)\text{,} \end{array}$$

where *g*_*t*_ indicates the output of the forget gate, *Z*_wg_, *Z*_rg_, and *Z*_dg_ are the weight matrices for the forget gate, and *a*_*g*_ is the variable bias of the forget gate.

The output gate of an LSTM neuron regulates how much current information of app review features is analyzed using equation (26). The contextual information of review features resulting from the output gate is defined as follows:

(26) $$\begin{array}{c} y_{t}=\sigma \left(S_{wy}w_{t}+S_{rw}r_{t-1}+S_{dy}d_{t}+a_{y}\right)\text{,} \end{array}$$

where *y*_*t*_ denotes the filtered information obtained from the output gate, *S*_wy_, *S*_ry_, and *S*_dy_ are the weight matrices for the output gate, and *a*_*y*_ is the variable bias of the output gate.

The state of the updated neuron or memory cell of LSTM is defined by

(27) $$\begin{array}{c} d_{t}=g_{t}d_{t-1}+x_{t}\tan\;h\left(S_{wd}w_{t}+S_{rd}r_{t-1}+a_{d}\right)\text{,} \end{array}$$

where *d*_*t*_ denotes the normalized situation of the updated neuron, *S*_wd,_ and *S*_rd_ are the weight matrices for the updated neuron, and *a*_*d*_ is the variable bias of the updated neuron.

The hidden state of the LSTM unit is defined by

(28) $$\begin{array}{c} r_{t}=y_{t}*\;\tan\;h\left(d_{t}\right)\text{,} \end{array}$$

where *r*_*t*_ is the hidden state of the LSTM unit at time *t*.

The BiLSTM unit combines the contextual information read by forward LSTM units and contextual information read by backward LSTM units. The output of the BiLSTM layer is defined by

(29) $$\begin{array}{c} r=\left[r_{forward},r_{backward}\right]\text{.} \end{array}$$

Following the BiLSTM layer, a dropout layer is introduced to reduce overfitting issues. The dense layer combines the outputs from the dropout layer. The output from the dense layer is presented to the sigmoid layer to predict the sentiment category of reviews using the following equation:

(30) $$\begin{array}{c} V=sigmoid\left(wr+b\right)\text{,} \end{array}$$

*V* denotes the prediction result for app reviews and *b* denotes the bias.

## 4. Results and Discussion

This section evaluates the performance of the proposed LSTM-DGSO model. The proposed method analyzes review sentiment categories by hyperparameter-optimized LSTM. The performance of classifying reviews into positive, negative, and neutral reviews by the LSTM-DGSO model is examined. The sentiment analysis performance of LSTM-DGSO is compared to existing sentiment analysis methods such as CNN, stochastic gradient descent (SGD), BiLSTM + attention mechanism, and SVM.

Accuracy is the proportion of accurately classified app reviews to the overall dataset count determined using the following equation:

(31) $$\begin{array}{c} Accuracy=\frac{TP+TN}{TP+TN+FP+FN}\text{.} \end{array}$$

TP indicates the count of negative app reviews identified exactly as negative. TN refers to the number of positive/neutral app reviews identified accurately as positive/neutral. FN indicates the number of negative app reviews misclassified as positive or neutral. FP denotes the number of positive/neutral app reviews misclassified as negative.

**Figure 6:**
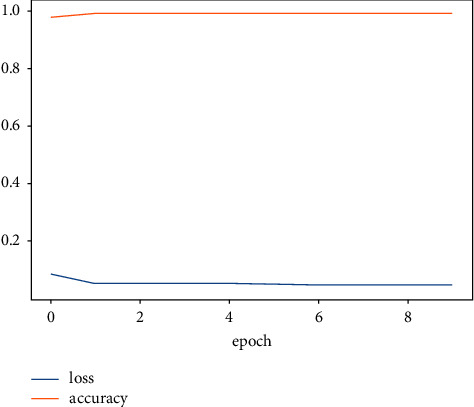
Overview of accuracy vs. loss.

The accuracy value indicates the number of correct predictions obtained. Loss values indicate the difference from the desired target sentiment categories.  Figure 6 portrays the overview of model accuracy versus loss. Once the model parameters are established, the model's accuracy is often measured as a percentage.

**Figure 7:**
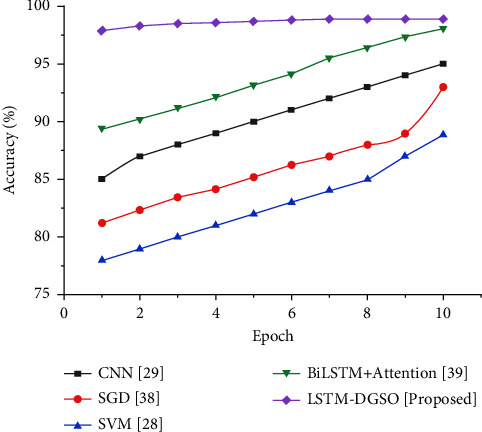
Comparative analysis epoch vs. accuracy for sentiment analysis of app reviews.

Figure 7 shows that the LSTM-DGSO model exhibited greater accuracy and lower loss for sentiment analysis. The proposed model's improved accuracy and lower loss portrayed the efficiency of the LSTM-DGSO model in review categorization into respective sentiment classes.

Precision is determined using (32) as the proportion of app reviews correctly identified as negative out of reviews identified as negative.

(32) $$\begin{array}{c} Precision=\frac{TP}{TP+FP}\text{.} \end{array}$$

**Figure 8:**
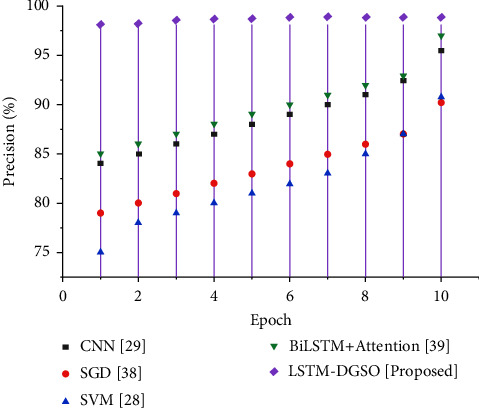
Comparative analysis epoch vs. precision for sentiment analysis of app reviews.

Figure 8 depicts the precision-based performance analysis of different sentiment analysis models. The precision of the LSTM-DGSO technique is higher than that of existing models. Higher precision implies that the number of positive/neutral app reviews misclassified as negative app reviews are low compared to that of existing models.

Recall is the proportion of app reviews correctly identified as negative out of the total negative reviews in the dataset and calculated by

(33) $$\begin{array}{c} Recall=\frac{TP}{TP+FN}\text{.} \end{array}$$

**Figure 9:**
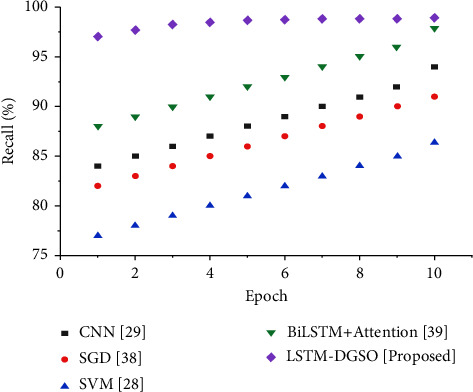
Comparative analysis epoch vs. recall for sentiment analysis of app reviews.

Figure 9 shows the recall-based performance analysis. The recall of the LSTM-DGSO technique is higher than that of existing sentiment analysis models, namely, CNN, SGD, BiLSTM + attention, and SVM. Higher recall for the proposed approach means that the number of negative app reviews misclassified as positive/neutral app reviews is low compared to that of existing models. A lower misclassification error achieved by the proposed model means that it can accurately identify the sentiment category of app reviews with low errors.

**Figure 10:**
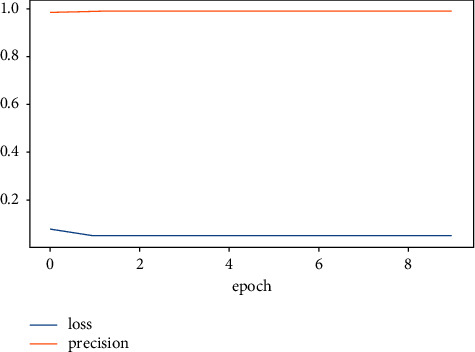
LSTM-DGSO model precision vs. loss.

**Figure 11:**
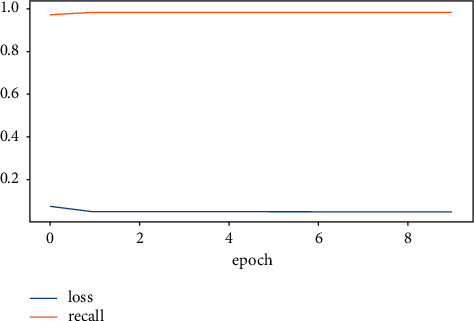
LSTM-DGSO model recall vs. loss.

Figures 10 and 11 represent the overview of model precision and recall versus loss, respectively. The LSTM-DGSO model revealed greater precision and recall and lower loss for app review analysis.

The F1 score is the weighted precision and recall ratio determined by

(34) $$\begin{array}{c} F1\;score=\frac{2*precision*recall}{precision+recall}\text{.} \end{array}$$

**Figure 12:**
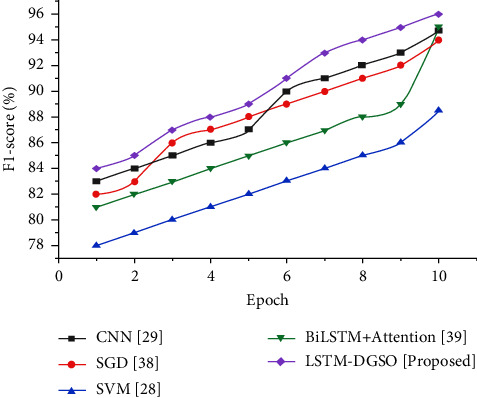
Comparative analysis epoch vs. the F1 score for sentiment analysis of app reviews.

Figure 12 depicts the comparative analysis of different sentiment analysis models based on the F1 score. The F1 score of the LSTM-DGSO technique is higher than that of the existing sentiment method considered in this study. A higher F1 score for the proposed approach indicates that the number of negative app reviews correctly classified as negative and the number of positive/neutral app reviews correctly classified as positive/neutral app reviews is significantly higher than those of existing models.

**Figure 13:**
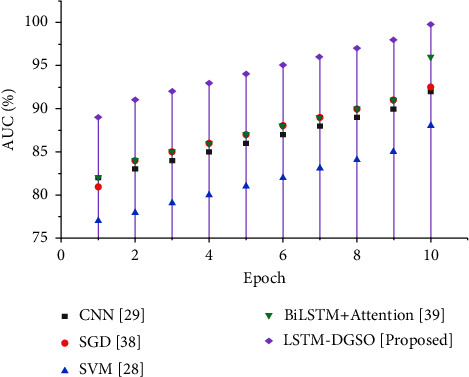
Comparative analysis epoch vs. AUC for sentiment analysis of app reviews.

The area under the curve (AUC) is used exclusively for probability-based classification issues to conduct in-depth prediction analysis.  Figure 13 illustrates the comparative analysis of different sentiment analysis models based on the AUC score.

**Figure 14:**
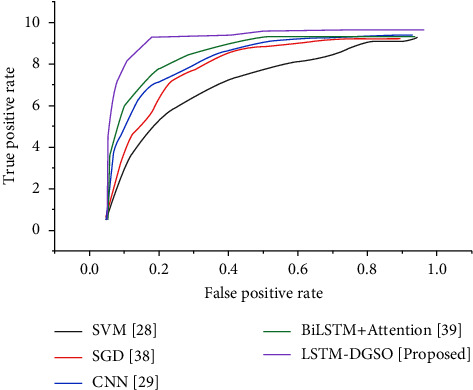
Comparative analysis ROC for various sentiment analysis models.

Figure 14 depicts the ROC curve for various sentiment analysis methods. The ROC curve illustrates the trade-off between sensitivity and specificity. The AUC score of the LSTM-DGSO technique is higher than that of existing sentiment analysis models. The LSTM-DGSO model's improved accuracy and lower error rate demonstrate the proposed model's robustness and convergence in sentiment analysis of reviews.

The comparative performance analysis with existing studies is illustrated in Table 4, and with different datasets [49–52] in Table 5. Training accuracy exhibits the classification performance of the LSTM-DGSO model for the training review dataset, and validation accuracy indicates the classification performance of the LSTM-DGSO model for the validation review dataset.

**Figure 15:**
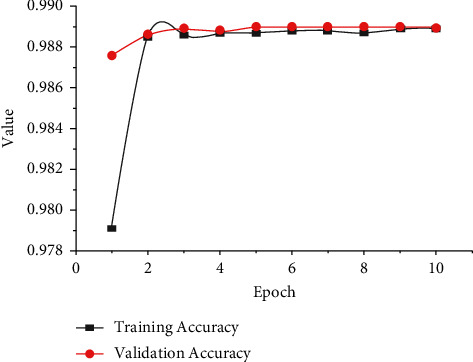
Comparative analysis of LSTM-DGSO model training and validation accuracies.

Figure 15 portrays the comparative investigation of training and validation accuracies for the proposed LSTM-DGSO model. The training set is the most significant subset formed from the original dataset and utilized to fit models. This subset is used to train models. The models are then evaluated based on their performance based on the validation set to complete the model selection process. From the analysis, it is observed that validation accuracy is slightly lower than training accuracy. The proposed model efficiently classifies the reviews into positive, negative, and neutral in the training and validation phases.

**Figure 16:**
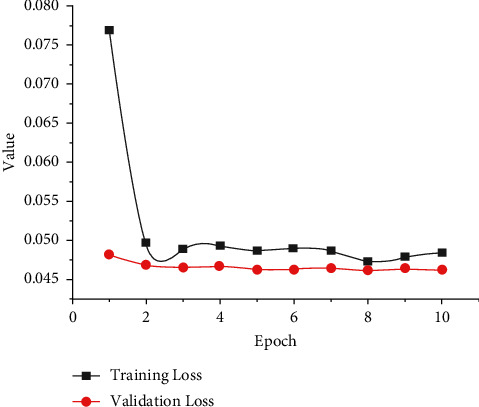
Comparative analysis of LSTM-DGSO model training and validation losses.

Figure 16 exhibits the comparative investigation of training and validation losses for the proposed LSTM-DGSO model. From the figure, it is observed that validation loss was slightly lower than training loss. It demonstrated that the proposed model fits the training and validation review datasets well.

Table 6 depicts the accuracy and loss indicated by LSTM-DGSO over training and validation review data and demonstrates that the LSTM-DGSO model efficiently mitigates overfitting issues and generalization errors. The proposed approach overcomes existing approaches, such as CNN, SGD, SVM, and Bi-LSTM. The CNN model did not accurately encode objectlocation, orientation, and a large amount of training data.o .The SVM approach is inappropriate for handling massive data sets, whereas SGD models can be reasonably computationally complex.

Figure 6 illustrates the accuracy-based performance analysis of different sentiment analysis models. As the epoch increases, the accuracy for the classification of reviews slightly increases for the proposed LSTM-DGSO model. The accuracy of the LSTM-DGSO technique is higher than that of existing sentiment analysis models, namely, CNN [29], SGD [36], BiLSTM + attention [37], and SVM [28]. Table 4 illustrates the performance analysis of the proposed and conventional sentiment analysis models. In addition, the performance analysis of the proposed and conventional sentiment analysis models compared to other datasets like book reviews, IMDb movie reviews, Sentiment 140, and SemEval-2017 datasets is illustrated in Table 5 due to GA-based feature extraction and FA-based feature selection. The optimal review features selected by the FA efficiently improved the sentiment analysis.

Sentiment analysis can be affected by the effectiveness of the labeled datasets utilized, and construct validity is at threat. A decrease in the accuracy of sentiment analysis can be due to inconsistent annotation. The issue has been addressed using the BERT model to label the dataset and enhance the learning effectiveness of the model. Internal elements like how the proposed LSTM model hyperparameters are set up pose a threat to internal validity. The optimized LSTM model is used, obtained by theproposed DGSO method, to overcome the issue. 

## 5. Conclusion

The sentiment analysis posted by mobile app users is significant and delivers accurate insights into the app. This research employed an optimized DL model named LSTM-DGSO for sentiment analysis of online reviews. Effective feature extraction using the GA and feature selection employing the FA are used. The proposed LSTM-DGSO models demonstrated an accuracy of 98.89% and a loss of 0.0484 compared with existing conventional sentiment analysis methods.

The standard GWO cannot seamlessly transition from prospective exploration to exploitation by adding more iterations. The GWO's primary shortcoming is that its single search technique hinders its ability to manage optimization issues with varying characteristics competently. Traditional LSTM parameters are prone to falling into local optimum when traditional LSTM parameters are adjusted backward. The high complexity of the algorithm is a disadvantage that reduces prediction accuracy. Future research is needed for classifying reviews using DL optimization techniques based on multiple aspects, like satisfied/unsatisfied, like/dislike, and recommended/not recommended with different sets of datasets.

## Figures and Tables

**Algorithm 1:**
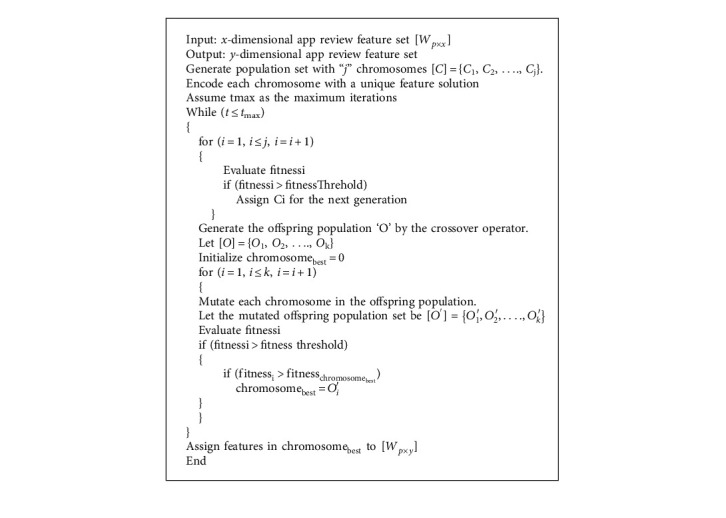
Genetic algorithm-based feature extraction.

**Algorithm 2:**
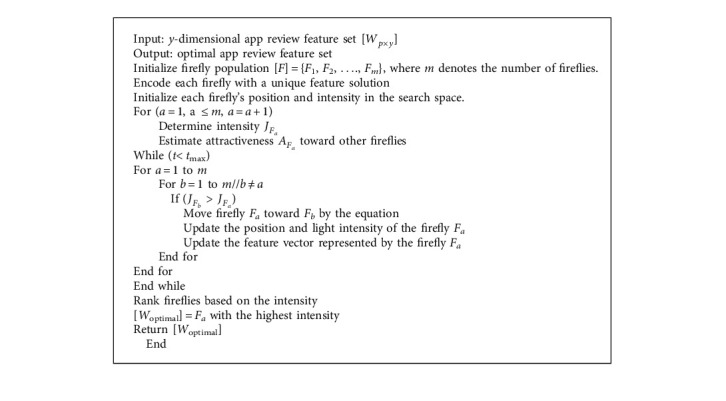
Firefly algorithm-based feature selection.

**Algorithm 3:**
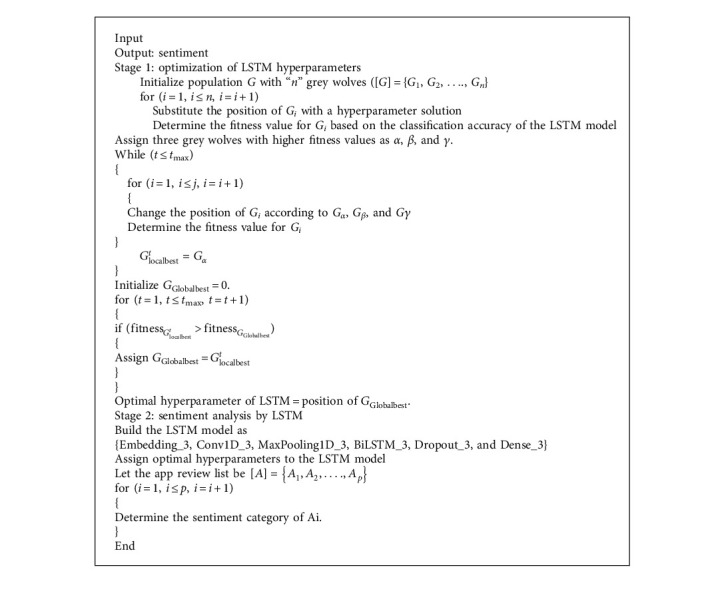
LSTM-DGWO-based sentiment analysis.

**Table 1:** Summary of existing sentiment analysis studies.

S.No	Reference	Technique	Advantage	Limitation
1	[14]	MaxEnt-JABST	Efficient for opinion extraction	Possess cross-domain sentiment analysis issues
2	[15]	NB and MaxEnt models	Carry out sentiment analysis at abstract levels	Less accuracy
3	[18]	DBNSA	Effectively classification depending on user ratings	Computationally complex
4	[19]	Neural network model	Efficient for binary sentiment classification	Extracted features do not suit nonbinary sentiment classification tasks
5	[21]	AT-MC-BiGRU-capsule	Used a capsule mechanism for text characterization	Stability issues
6	[24]	RNN	Effective even for larger data	Glove feature extraction resulted in lower accuracy
7	[25]	Deep CNN-LSTM	Efficient for review analysis in the e-commerce domain	Requires higher computational power
8	[30]	Optimized dictionary-based multilabel classification	Dynamically categorizes nonfunctional needs from app user feedback	Less efficiency
9	[33]	RF, SVM, and NB	Analyzes textual reviews of digital payment apps	Cost-inefficient
10	[29]	CNN	Effective classification of app reviews	Feature extraction is not efficient

**Table 2:** Dataset description.

Field name	Description	Example
app_id	Unique id for the app reviews	8ff58c99-e85c-44cd-ad13-7df88fbab704
Author	Title of the author	morÃ©
Rating	Count of stars allotted to the app by the author	5
posted_at	Date when the app user posts the review	June 2, 2022
Review	Body of the review	Great app. It has very good automation, which makes money 24/7. The support team is very helpful; thanks a lot to Lazar L.
helpful_count	The count of times the review was considered useful.	0
Developer_reply	Reply provided by the app developer for the reviews	—
Developer_reply_posted_at	Date when the app developer posts the reply	—

**Table 3:** The architecture of the proposed LSTM model.

Layer (type)	Output shape	Parameters
embedding_3 (embedding)	(None, 978, 32)	160000
conv1d_3 (Conv1D)	(None, 978, 32)	3104
max_pooling1d_3 (MaxPooling1D)	(None, 489, 32)	0
bidirectional_3 (BiLSTM)	(None, 64)	16640
dropout_3 (dropout)	(None)	0
dense_3 (dense)	(None)	195

**Table 4:** Performance analysis of various sentiment analysis models.

Model name	Dataset used	Accuracy (%)	Precision (%)	Recall (%)	F1 score (%)	AUC (%)
CNN [29]	Apple app store review	95	95.49	93.94	94.71	92
SVM [28]	Mobile app review	88.9	90.8	86.4	88.5	88
SGD [36]	Google Play app review	93	90.2	91	94	92.5
BiLSTM-attention [37]	Online car-hailing app review	98.6	97	97.9	95	96
LSTM-DGSO (proposed)	Shopify app review	98.89	98.89	98.89	96	99.7

**Table 5:** Performance analysis of various sentiment analysis models with other datasets.

Model name	Dataset used	Accuracy (%)	Precision (%)	Recall (%)	F1 score (%)	AUC (%)
SentiXGBoost [49]	SemEval-2017	90.8	92.7	98.1	94	93
GCNN + LSTM + SVM [50]	IMDb movie review	91.3	91.9	94	93.56	94.65
LSTM without embedding [51]	Sentiment140	96	93	95	95.67	9
SLCABG [7]	e-commerce book review	93.5	93	93.6	93.3	93.68
LSTM-DGSO (proposed)	Shopify app review	98.89	98.89	98.89	96	99.7

**Table 6:** Comparative analysis of LSTM-DGSO model efficiency in training and validation phases.

Epoch	Accuracy		Loss	
	Training accuracy	Validation accuracy	Training loss	Validation loss
1	0.9791	0.9876	0.077	0.0482
2	0.9885	0.9886	0.0497	0.0469
3	0.9886	0.9889	0.0489	0.0466
4	0.9887	0.9888	0.0493	0.0467
5	0.9887	0.989	0.0487	0.0463
6	0.9888	0.989	0.049	0.0463
7	0.9888	0.989	0.0487	0.0465
8	0.9887	0.989	0.0473	0.0462
9	0.9889	0.989	0.0479	0.0464
10	0.9889	0.989	0.0484	0.0462

## Data Availability

Data are taken from the below website and duly cited in reference [46] (https://www.kaggle.com/datasets/usernam3/shopify-app-store?select=reviews.csv).
